# Chromosome-level dataset from *de novo* assembly of a Fabada common bean genotype using Illumina and PacBio technologies

**DOI:** 10.1016/j.dib.2025.112219

**Published:** 2025-10-29

**Authors:** Jurado María, Die José Vicente, Ferreira Juan José, Campa Ana

**Affiliations:** a Plant Genetic Group, Regional Service for Agrofood Research and Development (SERIDA), 33300 Villaviciosa, Asturias, Spain; b Department of Genetics-ETSIAM, University of Córdoba, Campus de Rabanales, 14071, Córdoba, Spain

**Keywords:** *Phaseolus vulgaris*, Legume, *De novo* assembly, PacBio

## Abstract

Fabada is a traditional market class of common bean (Phaseolus vulgaris L.) cultivated in northern Spain, recognized for its distinctive seed phenotype with very large, white, and oblong seeds. High-throughput genotyping of this market class revealed that its genome is predominantly of Andean origin, with approximately 30 % introgression from the Mesoamerican gene pool. The de novo genome assembly of the A25 bean genotype, derived from the local cultivar ‘Andecha’ which belongs to the Fabada market class, is described. Two complementary technologies were used: PacBio (Sequel II) for long reads and Illumina (NovaSeq PE150) for short reads. The depth of coverage achieved was 112x for Illumina and 47x for PacBio. Genome assembly resulted in 468,9 Mbp (98.5 % BUSCO completeness), organized into 1363 scaffolds plus the mitochondrial and chloroplast genomes. Based on the reference genome (NCBI accession number GCF000499845.2), these scaffolds were organized into 11 chromosomes and 169 unplaced scaffolds. The mitochondrial genome was assembled based on that of P. vulgaris (NCBI accession number NC_045135). Two mitochondrial scaffolds were obtained, one of 371,437 bp, and the other of 11,183 bp. The chloroplast genome was assembled based on that of *P. vulgaris* (NCBI accession NC_009259.1), resulting in 161,310 bp. To the best of our knowledge, this is the first available genome of a common bean accession exhibiting recombination between the two major gene pools, Andean and Mesoamerican, and the second assembled genome of a European common bean.

Specifications Table

**Table:** 

Subject	Biology
Specific subject area	Genomics, Plant Sciences
Type of data	Genomic sequences in fasta files: PvulA25_chromosomes.fasta: chromosome level organization according to the v2 reference genome. - PvulA25_unmapped-scaffolds.fasta: unmapped scaffolds according to the v2 reference genome - PvulA25_Cp.fasta: chloroplast assembly. - PvulA25_Mt.fasta: mitocondrial assembly.
Data collection	*Genomic DNA was isolated from a pool of young trifoliate leaves of the line A25 (market class fabada) from 3–4-week-old plants. Sequencing was performed using the PacBio Sequel II system (long reads) and the Illumina NovaSeq PE150 (short reads).*
Data source location	*Plants were grown at the Regional Service for Agrofood Research and Development (SERIDA), Villaviciosa, Principality of Asturias, Spain (43°29′01́''N, 5°26′11′'W, elevation 6.5* m*).*
Data accessibility	*This Whole Genome Shotgun project has been deposited at DDBJ/ENA/GenBank under the accession JBNPQC000000000. The version described in this paper is version JBNPQC010000000.* *Repository name*: NCBI Data identification number: *JBNPQC000000000* Direct URL to data: https://www.ncbi.nlm.nih.gov/datasets/genome/GCA_051048075.1/
Related research article	*None*

## 1 Value of the Data

• This is the unique genome assembly available for the common bean market class Fabada.
• This genome is particularly valuable for studying and validating genes involved in seed size and quality, traits that exhibit extreme values in this market class.
• This is the second available genome of European origin, offering a new resource for studying the evolution of this species in Europe.
• This genome is valuable for completing the *P. vulgaris* pangenome.

## 2 Background

Common bean (*Phaseolus vulgaris* L.) is one of the most important food legumes for human consumption globally [1]. It is a highly diverse species, consisting of two main gene pools, Andean (A) and Mesoamerican (MA), which were independently domesticated in at least two parallel domestication events [2]. Both gene pools were successfully introduced and disseminated across Europe, making it possible to identify local European varieties with either A or MA gene pools, or with varying levels of introgression between them [3]. This is the case for the Fabada bean market class, whose genome is primarily of A origin with approximately 30 % introgression from the MA gene pool [4]. Fabada is a dry bean market class first described in northern Spain in the mid-20th century. It is characterized by a distinct seed phenotype, featuring very large white seeds (90–100 g/100 seeds) with an oblong shape and a length/width ratio greater than 2.2.

This study aimed to provide a newly sequenced genotype of the Fabada market class. It represents the second available genotype of European origin and, to the best of our knowledge, the first sequenced genotype displaying introgression between both gene pools of the species.

## 3 Data Description

Table 1 shows the standard metric parameters obtained from PacBio and Illumina sequence data.

**Table 1:** Standard metric parameters of PacBio and Illumina sequence data.

	N° reads	N° bases (bp)	Mean read length (bp)	N50
CCS (PacBio)	4459,685	81,154,094,113	18,197	20,657
HiFi (PacBio)	1831,809	34,429,383,781	18,795	18,917
Illumina (R1 + R2)	403,708,348	60,556,252,200	150	n/a

Based on the distribution of the k-mer sequences, the k-mer size parameter was set to *k* = 21(Figure S1).

The PacBio high fidelity (HiFi) reads and paired end 150 bp generated an initial assembly of 469,698 Mbp, which implies a 46.6x mean coverage for PacBio and 112x mean coverage for Illumina. The BUSCO quality parameters for this assembly were 98.5 % completeness, including 96.7 % single-copy and 1.8 % duplicated genes, with 0.2 % fragmentated and 1.3 % missing.

Mitochondrial and chloroplast reads were extracted using the corresponding organellar reference sequences for this species (NCBI Reference Sequence: NC_009259.1 and NC_045135, respectively). From these reads the organellar genomes where *de novo* assembled. The mitochondrial genome was organized into two scaffolds of 371,437 bp and 11,183 bp. The chloroplast genome yielded a circular molecule of 161,310 bp. The remaining nuclear reads were newly assembled based on paired-end RNA reads from seedling stage of the line A25 resulting in 1363 scaffolds. Quality metrics of these new assemblies are shown in Table 2.

**Table 2:** Quality metrics of the mitochondrial, chloroplast and scaffolded nuclear assemblies computed by QUAST.

Assembly	Mitochondrial	Chloroplast	Scaffolded nuclear
# contigs	2	1	1363
Largest contig	371,437	161,310	8759,870
Total length	382,620	161,310	469,287,317
GC ( %)	45.05	34.95	34.41
N50	371,437	161,310	1036,503
N90	371,437	161,310	152,353
auN	360,907.7	161,310.0	1696,220.7
L50	1	1	104
L90	1	1	570

The nuclear scaffolds were organized on chromosomes based on the v2 reference genome of the species (NCBI accession GCF_000499845.2). A total of 464,013,519 bp were organized in 11 chromosomes and 5,392,098 bp, comprising 169 scaffolds, were unplaced. Table 3 indicate the size of each chromosome and the GC % content.

**Table 3:** Chromosome-level assembly of the A25 genome (NCBI accession: GCA_051048075.1) based on the v2 reference genome of Phaseolus vulgaris (NCBI: GCF_000499845.2).

Chromosome	GeneBank	Size (bp)	GC %
1	CM117453.1	48,847,685	34.5
2	CM117454.1	50,204,780	33.5
3	CM117455.1	47,382,235	33.0
4	CM117456.1	41,649,249	35.0
5	CM117457.1	36,499,157	35.5
6	CM117458.1	28,425,830	33.0
7	CM117459.1	38,057,127	33.5
8	CM117460.1	53,820,110	35.0
9	CM117461.1	38,145,473	32.5
10	CM117462.1	35,611,486	36.0
11	CM117463.1	45,370,387	35.5

The quality metrics of this chromosome assembly computed by QUAST revealed a N50 value or 45,370,387 bp, L50 value of 5, with a gap percentage of 0.026 %. The BUSCO quality parameters for this assembly were 98.0 % completeness, including 95.0 % single-copy and 3.0 % duplicated genes, with 0.7 % fragmentated and 1.3 % missing.

**Fig. 1:**
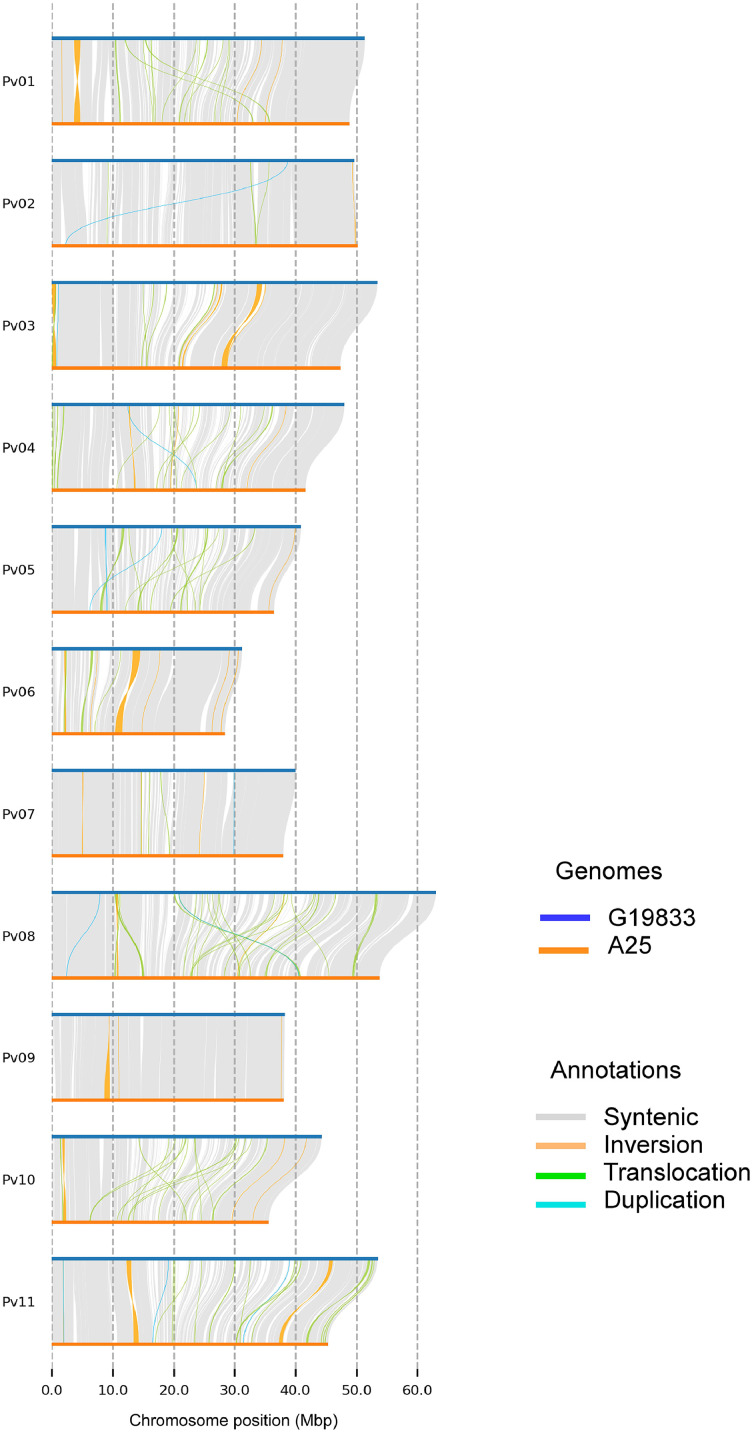
Genome-wide comparison of structural variants between the reference *P. vulgaris* (GCF_000499845.2) and A25 (GCA_051048075.1) genomes. Syntenic regions, inversions, translocations, and duplications are displayed across the 11 chromosomes.

The QUAST comparison between the A25 genome and the reference one shows an unaligned length of 28.8 Mbp and a genome fraction covered of 81,97 %, reflecting moderate completeness. Fig. 1 illustrates a comprehensive genome-wide comparison between both genomes and it shows conserved blocks and structural variations. Chromosomes Pv02, Pv07, and Pv09 appear to be the most conserved between the two genomes, showing extensive syntenic regions and fewer structural variations. In contrast, a high frequency of structural variations, such as inversions, duplications, and translocations, is found in the remaining chromosomes. In addition, the figure shows the unaligned regions of the reference genome.

## 4 Experimental Design, Materials and Methods

### 4.1 Genomic DNA extraction and sequencing

Line A25 proceeds from a selection of the local variety ‘Andecha’, which has an indeterminate climbing growth habit, very large seeds (100 g per 100 seeds) and belongs to the Fabada market class (Figure S2). For DNA extraction, leaf tissue was frozen in liquid nitrogen and placed into a 2 mL round-bottom tube, previously chilled on dry ice, containing two stainless steel beads. The frozen tissue was ground together with the beads and SDS as the liquid medium using a TissueLyser LT (Qiagen). After lysis, proteins were precipitated using potassium acetate. DNA was then immobilized on magnetic beads for washing, and finally eluted in 50 µL of elution buffer. DNA was quantified using the Qubit high-sensitivity dsDNA assay (Thermo Fisher Scientific). The genomic DNA libraries were constructed by the company Allgenetics & Biology S.L. following a previous protocol [5] with minor modifications for Illumina and the SMRTbell Express Template Prep Kit 2.0 for PacBio. The fragment size distribution and concentration of the libraries were determined using an Agilent 2100 Bioanalyzer using the Agilent HS DNA kit.

The Illumina library was sequenced by the company Allgenetics & Biology S.L. on an Illumina NovaSeq 6000 system with paired-end 150 bp reads (PE150), generating approximately 55 gigabases of dataThe PacBio library was sequenced on a Sequel II platform with SMRT (Single Molecule Real-Time) Cell 8 M and Circular-Consensus Sequencing (CCS) mode. The resulting CCS raw reads were converted to HiFi reads. Short Illumina reads were trimmed for adapters and filtered with Trimmomatic v 0.39 [6] using a minimum quality threshold of Q30, a minimum length of 50 bp, and quality trimming at the end with a cutoff below Q25. The quality of the reads obtained from the FASTQ files was assessed using FastQC v0.11.9.

### 4.2 Genome assembly

The KMC v3.1.1 [7] program was used to count the cases of different k-mers in quality-filtered reads. GenomeScope 2.0 [8] was used to visualize the fit of the k-mer profile to a model of the expected fractions corresponding to sequencing errors, single-copy, and multi-copy parts of the genome.

Short and long genomic sequencing reads were de novo assembled into mega-reads using MaSuRCA v3.4.2 [9], and subsequently polished using POLCA v4.1.0 [10]. The quality and completeness of the assembly were evaluated using BUSCO v5.beta.1 [11] and the embryophyta_odb10 database.

Organellar genome reads (mitochondrial and chloroplast) were detected by aligning all initial quality-filtered reads to the complete chloroplast and mitochondrial genomes of *P. vulgaris* (NCBI Reference Sequence: NC_009259.1 and NC_045135, respectively) using the BWA-MEM (0.7.15-r1140) algorithm. The mapped reads were extracted using SAMtools [12] and Sambamba [13] and used for *de novo* assembly in NOVOPlasty v4.2 [14] with the complete chloroplast and mitochondrial genomes used as the seed sequence, respectively. For the assembly of organellar genomes k-mer was set to 39 bp. The remaining nuclear reads were newly scaffolded based on RNA reads to improve the contiguity and the accuracy of the assembly. Paired-end RNA reads obtained from seedling stage of the line A25 (NCBI accession: PRJNA851559) were used in AGOUTI v0.3.3 [15]. The quality of the chloroplast, mitochondrial, and the newly generated nuclear scaffolded assembly were evaluated with the package QUAST 5.0.2 [16].

The new resulted nuclear assembly was organized in chromosomes using scaffold module of RagTag v2.1.0 [17] and the v2 reference genome of *Phaseolus vulgaris* (NCBI accession GCF_000499845.2). The quality of the chromosome assembly was checked using BUSCO and QUAST [12,17].

QUAST was used to perform a brief comparative analysis of the obtained nuclear assembly against the reference genome. Genome alignments between the two assemblies were performed using minimap2 [18]. Structural variants and rearrangements were detected using SyRi [19] and plotting using plotsr [20].

## Limitations

Not applicable.

## Ethics Statement

The authors have reviewed and adhered to the ethical guidelines for publication in Data in Brief and confirming that the current work does not involve human subjects, animal experiments, or data collected from social media platforms.

## CRediT authorship contribution statement

**Jurado María:** Data curation, Formal analysis, Methodology, Writing – original draft. **Die José Vicente:** Writing – review & editing. **Ferreira Juan José:** Conceptualization, Funding acquisition, Writing – review & editing. **Campa Ana:** Conceptualization, Methodology, Supervision, Funding acquisition, Writing – review & editing.

## Data Availability

National Center for Biotechnology InformationPhaseolus vulgaris Fabada: whole genome sequencing and assembly (Original data)

## Appendix Supplementary materials

**Figure S1.** GenomeScope k-mer profile and model fit plot for A) PacBio data and B) Illumina data, based on a k-mer size of 21. The observed k-mer frequency distribution, depicted in blue, represents the number of times a given k-mer was observed in the sequencing data (coverage), and the total number of k-mers with a given coverage (frequency). Image, image 1

**Figure S2.** Seeds of the A25 genotype included in the Fabada Market class featuring very large white seeds (90–100 g/100 seeds) with an oblong shape and a length/width ratio greater than 2.2. Each grid on the template measures 1 × 1 cm. Image, image 2
