# Supplementary figures and images for: Chromosome-level dataset from de novo assembly of a Fabada common bean genotype using Illumina and PacBio technologies
Source: Data Brief. 2025 Oct 29;63:112219. doi: 10.1016/j.dib.2025.112219 (PMC12666108; doi:10.1016/j.dib.2025.112219)

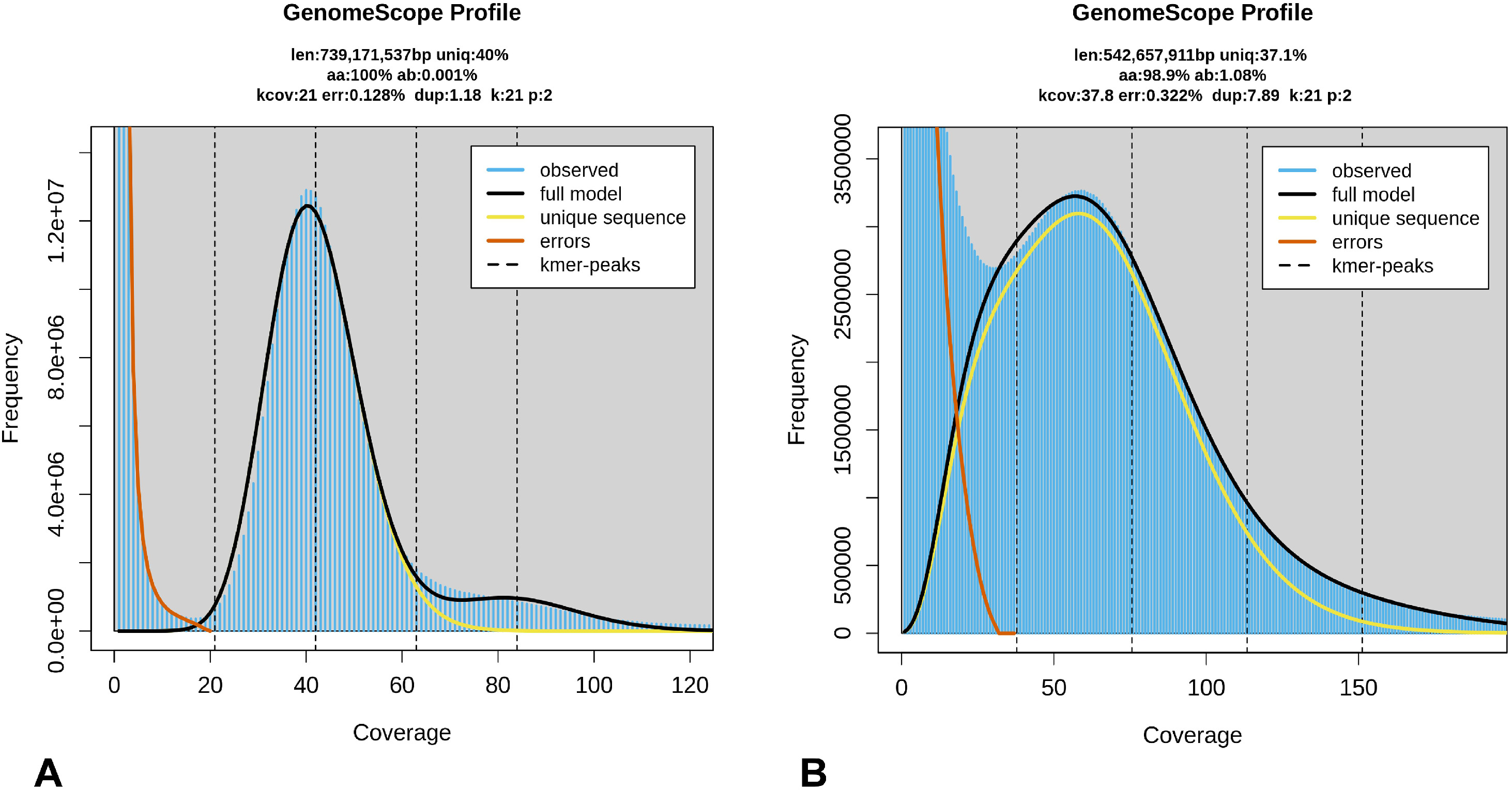

Supplement: Supplementary file 1 — Figure S1. GenomeScope k-mer profile and model fit plot for A) PacBio data and B) Illumina data, based on a k-mer size of 21. The observed k-mer frequency distribution, depicted in blue, represents the number of times a given k-mer was observed in the sequencing data (coverage), and the total number of k-mers with a given coverage (frequency). [file mmc1.jpg]

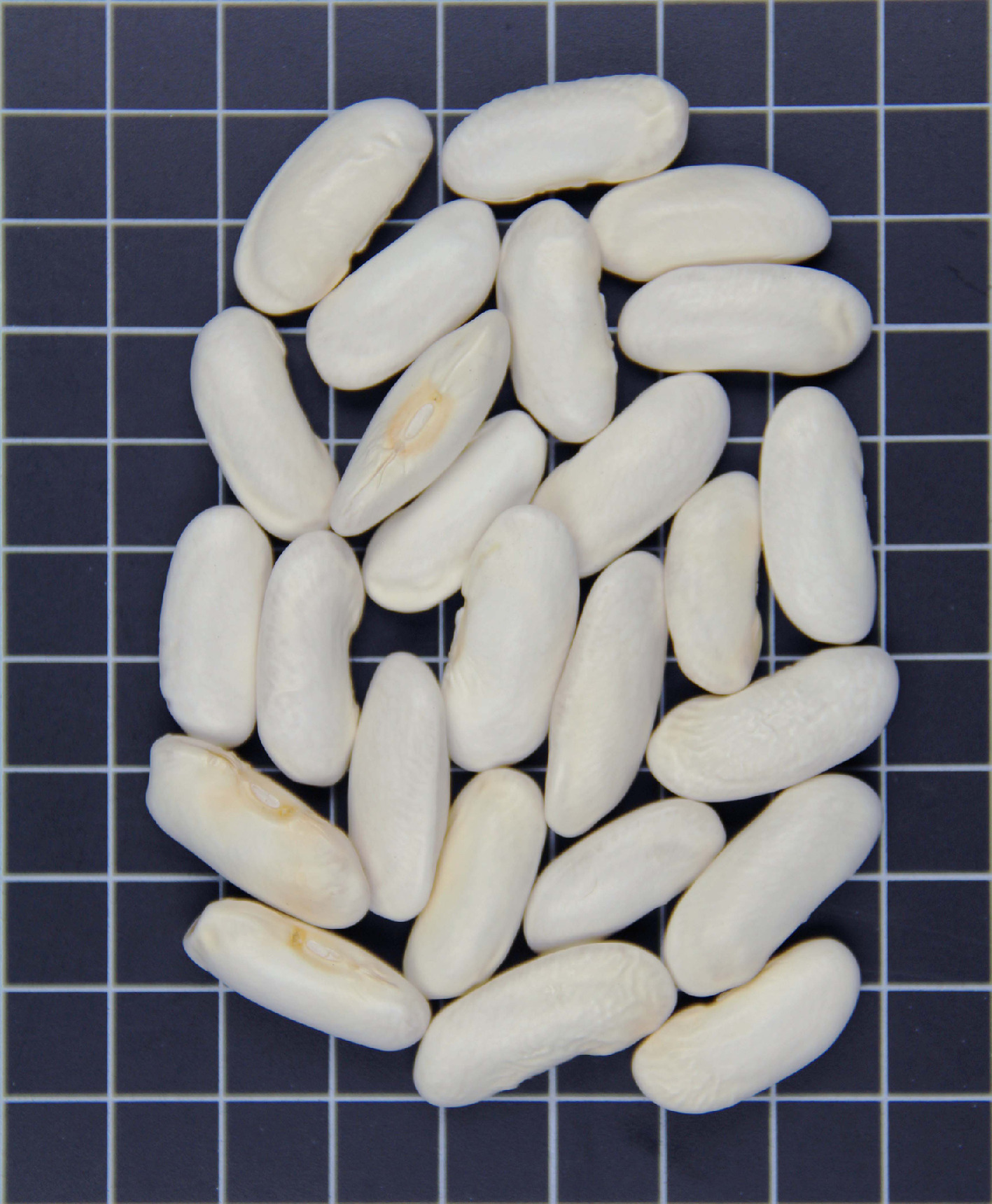

Supplement: Supplementary file 2 — Figure S2. Seeds of the A25 genotype included in the Fabada Market class featuring very large white seeds (90–100 g/100 seeds) with an oblong shape and a length/width ratio greater than 2.2. Each grid on the template measures 1 × 1 cm. [file mmc2.jpg]
